# Shake flask methodology for assessing the influence of the maximum oxygen transfer capacity on 2,3-butanediol production

**DOI:** 10.1186/s12934-019-1126-9

**Published:** 2019-05-03

**Authors:** Benedikt Heyman, Robin Lamm, Hannah Tulke, Lars Regestein, Jochen Büchs

**Affiliations:** 1 RWTH Aachen University, AVT–Biochemical Engineering, Forckenbeckstraße 51, 52074 Aachen, Germany; 2 Leibniz Institute for Natural Product Research and Infection Biology, HKI Beutenbergstraße 11a, 07745 Jena, Germany

**Keywords:** *Bacillus licheniformis*, Oxygen availability, Oxygen transfer rate, Respiratory quotient, 2,3-Butanediol, Acetoin, Scalability, Microaerobic conditions, Anoxic, Online monitoring

## Abstract

**Background:**

Production of 2,3-butanediol from renewable resources is a promising measure to decrease the consumption of fossil resources in the chemical industry. One of the most influential parameters on biotechnological 2,3-butanediol production is the oxygen availability during the cultivation. As 2,3-butanediol is produced under microaerobic process conditions, a well-controlled oxygen supply is the key parameter to control biomass formation and 2,3-butanediol production. As biomass is on the one hand not the final product, but on the other hand the essential biocatalyst, the optimal compromise between biomass formation and 2,3-butanediol production has to be defined.

**Results:**

A shake flask methodology is presented to evaluate the effects of oxygen availability on 2,3-butanediol production with *Bacillus licheniformis* DSM 8785 by variation of the filling volume. A defined two-stage cultivation strategy was developed to investigate the metabolic response to different defined maximum oxygen transfer capacities at equal initial growth conditions. The respiratory quotient was measured online to determine the point of glucose depletion, as 2,3-butanediol is consumed afterwards. Based on this strategy, comparable results to stirred tank reactors were achieved. The highest space–time yield (1.3 g/L/h) and a 2,3-butanediol concentration of 68 g/L combined with low acetoin concentrations and avoided glycerol formation were achieved at a maximum oxygen transfer capacity of 13 mmol/L/h. The highest overall 2,3-butanediol concentration of 78 g/L was observed at a maximum oxygen transfer capacity of 4 mmol/L/h.

**Conclusions:**

The presented shake flask approach reduces the experimental effort and costs providing a fast and reliable methodology to investigate the effects of oxygen availability. This can be applied especially on product and by-product formation under microaerobic conditions. Utilization of the maximum oxygen transfer capacity as measure for the oxygen availability allows for an easy adaption to other bioreactor setups and scales.

**Electronic supplementary material:**

The online version of this article (10.1186/s12934-019-1126-9) contains supplementary material, which is available to authorized users.

## Background

2,3-Butanediol is a widely used platform chemical with numerous applications in the chemical and pharmaceutical industry. Amongst others, it is used for 1,3-butadiene or methyl-ethyl-ketone synthesis and is a basis for several resins, solvents, fuels, polymers, food additives, plasticizers, cosmetics and drugs [1–4]. As bivalent alcohol with two chiral centers, three stereoisomers of 2,3-butanediol exist, two optically active isomers (d(−)- and l(+)-2,3-butanediol) and the optically inactive meso-2,3-butanediol. However, in many applications achiral products like 1,3-butadiene or methyl-ethyl-ketone are derived from 2,3-butanediol and the stereo-confirmation does not affect the conversion [5].

Conventionally, 2,3-butanediol is produced via chemical synthesis. Nevertheless, due to ecological as well as political concerns and to decouple the 2,3-butanediol price from fluctuating oil prices, biotechnological 2,3-butanediol production has been focused in many research activities, which have been summarized in numerous review articles [1–3, 6–9]. Production of high 2,3-butanediol concentrations up to 150 g/L has been reported especially with pathogenic organisms like *Klebsiella pneumoniae* [10], *Klebsiella oxytoca* [11], *Enterobacter cloacae* [12], *Enterobacter aerogen*es [13] and *Serracia marcescens* [14]. However, utilization of pathogenic organisms results in higher safety regulations and, therefore, higher costs in industrial applications. Consequently, non-pathogenic 2,3-butanediol producers are promising candidates for industrial production processes [15, 16]. Genetic engineering enables 2,3-butanediol production with well-characterized strains like *Escherichia coli* [17–20], *Saccharomyces cerevisiae* [21, 22], *Zymomonas mobilis* [23] and *Corynebacterium glutamicum* [24, 25]. Furthermore, some *Bacillus* and *Paenibacillus* strains naturally produce 2,3-butanediol [15, 26]. Especially, high 2,3-butanediol concentrations of 145 g/L with a space–time yield of 1.14 g/L/h were achieved with the wild type *Bacillus licheniformis* DSM 8785 by additional glucose pulsing [15]. These performance parameters are in the range of the ones of frequently used pathogenic organisms [2].

Independent of the chosen organism, the oxygen availability during the cultivation is generally accepted to be one of the most influential parameters for 2,3-butanediol production, which occurs under oxygen-limited or microaerobic conditions. The 2,3-butanediol yield increases at lower oxygen availability, while cell growth is favored at high oxygen availability, resulting in faster product formation rates [27, 28]. Furthermore, also the production of by-products strongly depends on the availability of oxygen. Accumulation of acetoin is observed at high oxygen availability, whereas glycerol and ethanol are formed at low oxygen availability [29, 30].

Several different process strategies have been developed to improve 2,3-butanediol production depending on the oxygen availability. Zeng et al. [31] used the respiratory quotient (RQ) as control parameter and Beronio and Tsao [32] maintained constant biomass specific oxygen uptake rates. The contrarious effects of oxygen availability on growth and product formation have been addressed in various studies by the application of two-stage cultivation strategies to accelerate 2,3-butanediol production [11, 14, 33–39]. Hereby, an initial phase of high oxygen availability leads to increased cell growth and, afterwards, 2,3-butanediol production is favored at lower oxygen availability. However, most studies defined the oxygen availability solely based on variations of reactor specific parameters like the agitation or aeration rate. Thus, the resulting oxygen availability and the observed effects are highly dependent on the utilized reactor geometry and operation. To establish general cultivation strategies, detailed characterization and quantification of the influence of oxygen availability on 2,3-butanediol production is required. Thereby, scalable parameters like the k_L_a value or the maximum oxygen transfer capacity should be used to quantify the oxygen availability independently of the utilized reactor type, geometry and operation. Only a limited number of studies investigated the effect of oxygen availability based on these parameters [27, 30, 35, 40–42]. The studies based on the k_L_a value led to contradictory results, as the highest 2,3-butanediol concentration with *K. oxytoca* was obtained at 18 h^−1^ by Ji et al. [35] and at 78 h^−1^ by Ramachandran et al. [42]. This contradiction can be attributed either to different optimum oxygen availabilities amongst individual *K. oxytoca* strains, or to the different utilized dynamic methods for k_L_a determination (Na_2_SO_3_ method and gassing out method, respectively). The maximum oxygen transfer capacity leading to the highest 2,3-butanediol concentration differs between different organisms. The highest concentrations were reached at about 15 mmol/L/h for *P. polymyxa* and *K. pneumonia* [40, 41] and at 10 mmol/L/h for *B. licheniformis* and *K. oxytoca* [27, 30]. This demonstrates the need for a fast, inexpensive and reliable methodology to identify the influence of the oxygen availability on 2,3-butanediol production. A necessary prerequisite is the reliable quantification of the maximum 2,3-butanediol concentration. However, 2,3-butanediol is consumed by the organism upon substrate depletion [2]. Consequently, the maximum 2,3-butanediol concentration has to be determined exactly at this point. Otherwise, the difference between the sampling time and the time of substrate depletion hampers the interpretation of the experimental results. Furthermore, the previously mentioned studies on the effect of oxygen availability on 2,3-butanediol production were performed in stirred tank reactor cultivations, which generally require high cultivation volumes, technical effort and long setup times.

The aim of this work is the development of a shake flask methodology to increase the experimental throughput and to reduce the experimental effort and costs. Moreover, the gained results are scalable and transferable to other reactor systems. First, a defined two-stage protocol for the cultivation of *Bacillus licheniformis* DSM 8785 was developed. At the beginning of the cultivation, a high shaking frequency results in oxygen-unlimited conditions and consequently unrestricted biomass formation. At a defined oxygen transfer rate (OTR) different levels of oxygen limitation are initiated in shake flasks with different filling volumes. Afterwards, a procedure to determine the maximum 2,3-butanediol concentration at the time of substrate depletion has been developed. On this basis, the influence of the maximum oxygen transfer capacity on the cultivation was investigated. Thereby, a fast and reliable methodology to select the ideal cultivation conditions based on the overall demand of any individual process is presented.

## Results

**Fig. 1:**
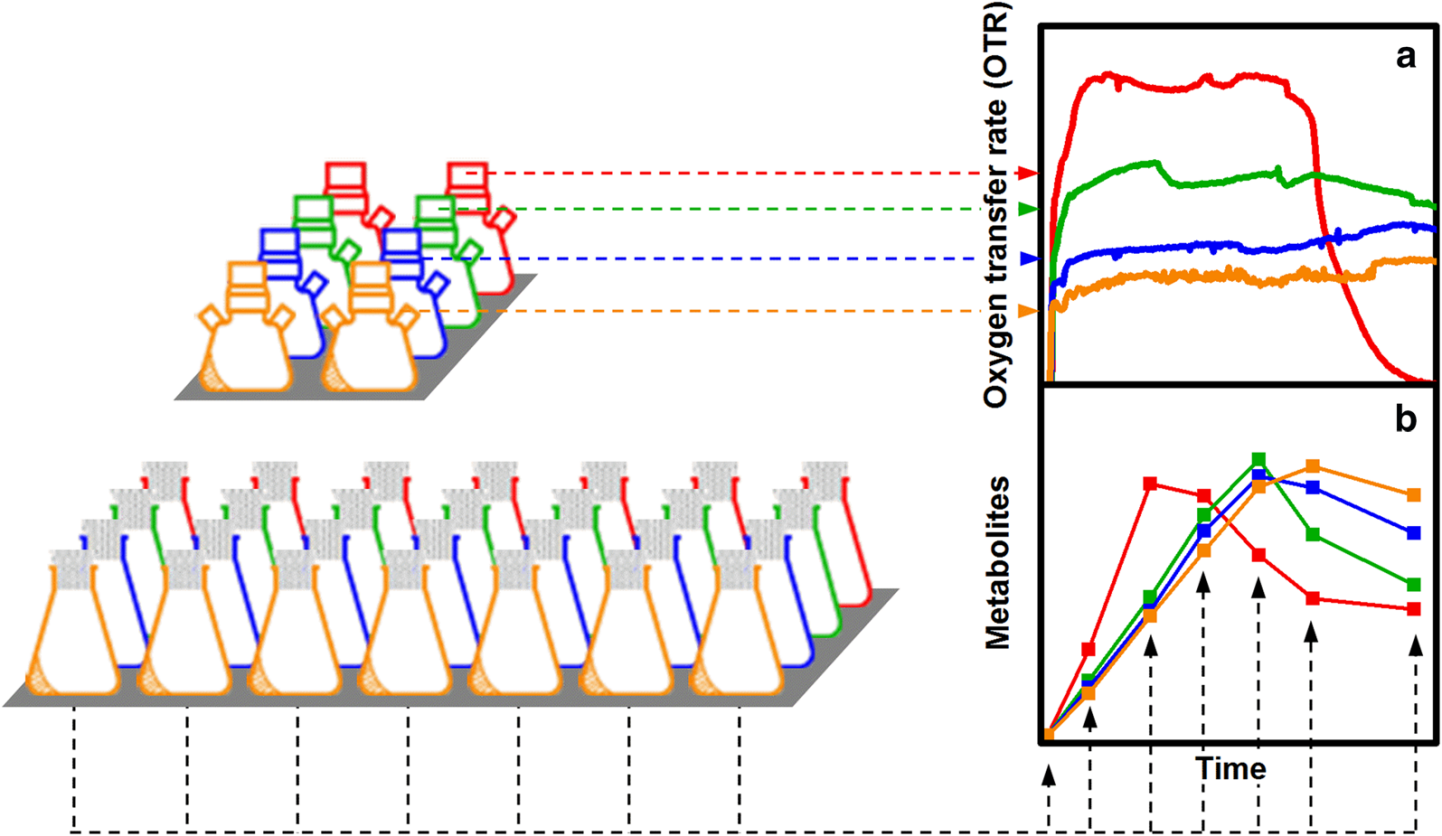
Schematic illustration of the experimental setup. Cultivations are performed in multiple individual shake flasks in parallel. As indicated by different colors, four different experimental conditions are investigated in one experiment. Online measurement (**a**) of oxygen transfer rate (OTR), carbon dioxide transfer rate (CTR) and the respiratory quotient (RQ) is performed as duplicates in a respiration activity monitoring system (RAMOS). Offline samples (**b**) are taken from individual shake flasks, which are not returned to the shaker after sampling

High 2,3-butanediol production with *B. licheniformis* has been demonstrated in cultivations in baffled shake flasks by Jurchescu et al. [15]. As 2,3-butanediol is produced under oxygen-limited conditions, only low maximum oxygen transfer capacities are required that can easily be achieved with unbaffled shake flasks. Furthermore, utilization of unbaffled shake flasks results in more defined flow regimes and reduces the experimental error [43, 44]. Therefore, cultivation of *B. licheniformis* was performed in unbaffled 250 mL shake flasks in this study. As visualized in Fig. 1, parallel cultivations were performed in multiple shake flasks. Oxygen and carbon dioxide transfer rates (OTR, CTR) and the respiratory quotient (RQ) were determined with the Respiration Activity Monitoring System (RAMOS) [45] (Fig. 1a). Parallel cultivations in conventional shake flasks were used for offline sampling (Fig. 1b). For each sampling point, one flask was removed from the shaker, subjected to offline analysis and not returned to the shaker. Consequently, cultivation data for each experimental condition are derived from up to nine individual shake flasks. High reproducibility is given when a steady course of all offline samples is observed.

**Fig. 2:**
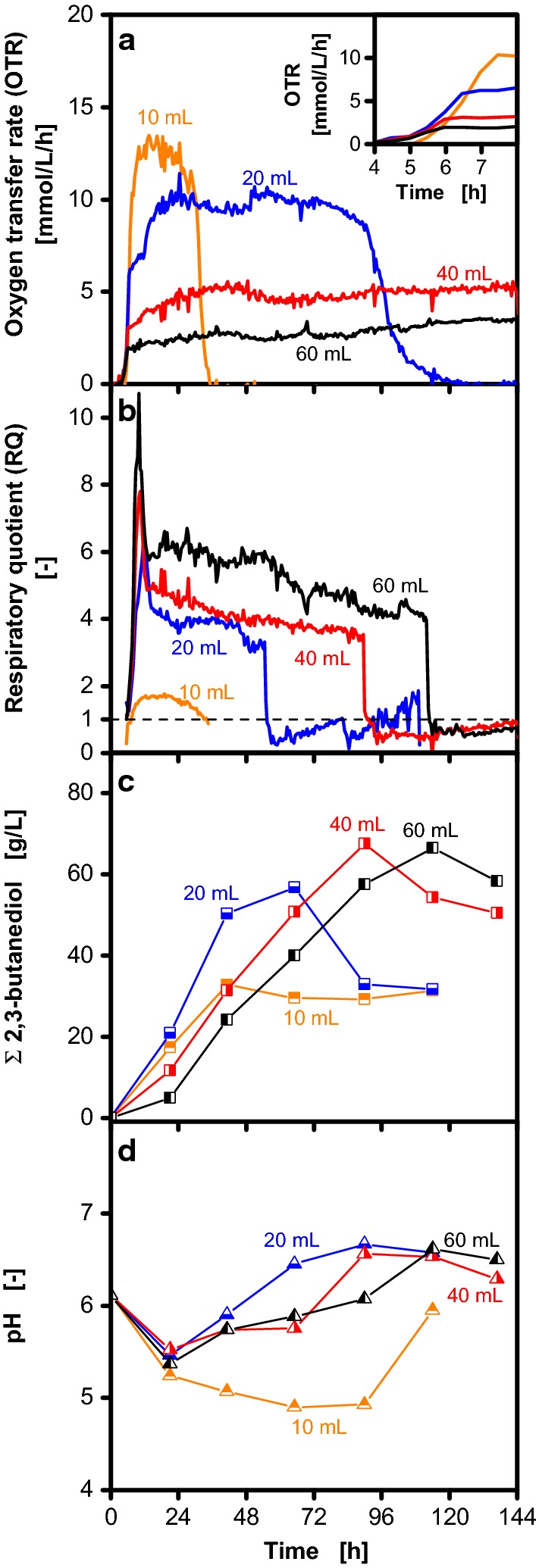
Cultivation of *Bacillus licheniformis* DSM 8785 under oxygen-limited conditions. Variation of the filling volume results in different maximum oxygen transfer capacities in shake flask cultivations. Data on oxygen transfer rate (OTR) (**a**), respiratory quotient (RQ) (**b**) total 2,3-butanediol concentration (**c**) and pH (**d**) are depicted. 2,3-Butanediol is shown as sum of the stereoisomers. The RQ is only shown for OTR > 1 mmol/L/h. To account for the increase of metabolite concentrations due to evaporation of water, all concentrations were corrected accordingly and referred to the initial filling volume. Cultivation conditions: 250 mL unbaffled shake flasks, temperature: 37 °C, shaking frequency: 100 rpm, shaking diameter: 50 mm, Nakashimada medium without addition of MES buffer

To investigate the effect of oxygen availability during the cultivation, different maximum oxygen transfer capacities can be adjusted by variation of the filling volume in shake flasks. In Fig. 2 cultivations with filling volumes between 10 and 60 mL are shown, which were performed in the same medium used by Jurchescu et al. [15]. For all filling volumes a steep increase of the OTR after the lag phase is followed by a plateau (Fig. 2a) indicating oxygen limitation [45]. The level of the plateau, which corresponds to the respective maximum oxygen transfer capacity, increases from 2.5 to 13 mmol/L/h with decreasing filling volume. The transition from unlimited growth to oxygen-limited conditions occurs as soon as the maximum oxygen transfer capacity is reached. Therefore, cultivations with low maximum oxygen transfer capacity (high filling volumes) have a shorter unlimited growth phase compared to cultivations with a high maximum oxygen transfer capacity (low filling volumes) as shown in the magnified inlet of Fig. 2a.

Further information about the metabolic activity of the cultures can be derived from the RQ (Fig. 2b). The RQ of the cultivation with 10 mL filling volume slowly increases to values around 2 and then drops together with the OTR after 30 h. For higher filling volumes, higher RQ levels can be observed. During the steep increase of the OTR, RQ values around 1 indicate aerobic cell growth. When oxygen-limited conditions are reached, the RQs increase and stay nearly constant at values between 4 and 6 for 50, 90 and 115 h (20, 40 and 60 mL, respectively). Hereby, lower maximum oxygen transfer capacities (higher filling volumes) result in higher RQs. The course of the RQ can be explained by 2,3-butanediol production (Fig. 2c), which leads to high RQs, as 2,3-butanediol is more reduced than the substrate glucose. Subsequent consumption of 2,3-butanediol results in RQ values below 1. The course of the pH during the cultivations is shown in Fig. 2d. At the beginning of the cultivation, the pH decreases due to the formation of organic acids and the consumption of ammonia [2, 46]. In the further course of the cultivation, the organism switches from organic acid to 2,3-butanediol formation to prevent further acidification [2] and the pH increases due to organic acid consumption. After 21 h the lowest pH is observed for the cultivation with 10 mL filling volume. At this condition, the pH further decreases to values below 5 and does not increase as observed for the other cultivations. This stronger acidification explains the deviation to the other cultivations, as it causes the metabolic activity to stop after 30 h (Fig. 2a, b) resulting in much lower overall 2,3-butanediol production (Fig. 2c).

The results presented in Fig. 2 reveal three obstacles for a precise assessment of the effects of varied maximum oxygen transfer capacities on 2,3-butanediol production. First, high maximum oxygen transfer capacities cannot be investigated, as the medium is not sufficiently buffered to prevent acidification. Second, the length of the unlimited growth phase at the beginning of the cultivation depends on the target maximum oxygen transfer capacity. Therefore, changes of the maximum oxygen transfer capacity do not solely affect the oxygen availability during the oxygen-limited phase, but also lead to inconsistent growth conditions. Third, 2,3-butanediol is consumed after the maximum concentration is reached. Therefore, a methodology to determine the maximum 2,3-butanediol concentration is required to properly compare different cultivation conditions.

**Fig. 3:**
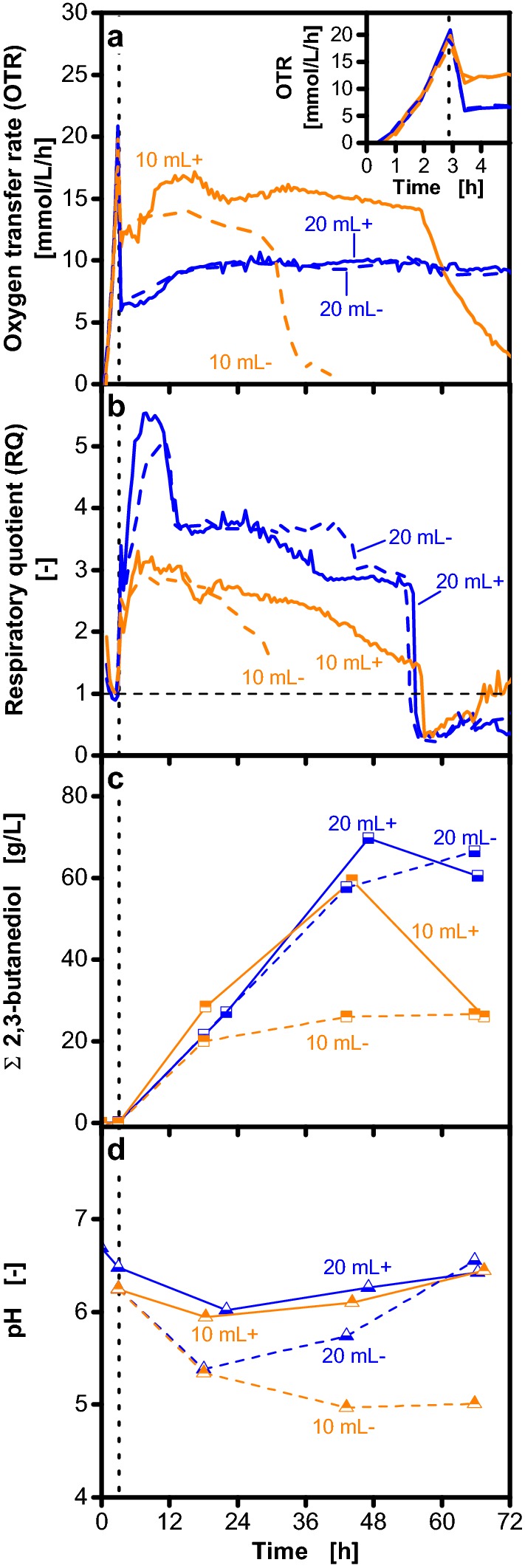
Effects of buffering on 2,3-butanediol production with *Bacillus licheniformis* DSM 8785. Cultivations on Nakashimada medium with (+) and without (−) addition of 100 mM MES buffer are compared. Variation of the filling volume results in different maximum oxygen transfer capacities in shake flask cultivations. As indicated by the vertical dotted line, the shaking frequency was reduced from 350 to 100 rpm after 3 h. Thereby, oxygen-limited conditions are induced at the same time for all cultivations. Data on oxygen transfer rate (OTR) (**a**), respiratory quotient (RQ) (**b**) total 2,3-butanediol concentration (**c**) and pH (**d**) are depicted. 2,3-Butanediol is shown as sum of the stereoisomers. The RQ is only shown for OTR > 1 mmol/L/h. To account for the increase of metabolite concentrations due to evaporation of water, all concentrations were corrected accordingly and referred to the initial filling volume. Cultivation conditions: 250 mL unbaffled shake flasks, temperature: 37 °C, shaking frequency: 350/100 rpm, shaking diameter: 50 mm

In the used cultivation medium, acidification cannot be prevented as the buffer capacity of the utilized phosphate buffer (with pK_a_ values of 2, 7.4 and 12.3) is below 10% at the observed pH values during the cultivation. To reduce acidification, an increased initial pH and addition of different MES buffer (pK_a_ = 6.1) concentrations was tested (Additional file 1). The increased initial pH did not prevent acidification and addition of 200 mM MES buffer slightly prolonged the cultivation. Therefore, 100 mM MES buffer was added to reduce the acidification during the cultivation in the following experiments (Fig. 3). However, tight pH control should be avoided as with decreasing pH the metabolism shifts from organic acid to 2,3-butanediol production [2]. Furthermore, a defined two-stage cultivation profile was applied in the experiment shown in Fig. 3. At the beginning of the cultivation, a shaking frequency of 350 rpm results in oxygen-unlimited conditions for all cultivations (Fig. 3a). This results in a steeply increasing OTR for all filling volumes. When cultivations reached an oxygen transfer rate of 20 mmol/L/h, the shaking frequency was reduced to 100 rpm to induce oxygen limitation. By this two-stage cultivation profile, defined and comparable cultivation conditions are obtained. The maximum oxygen transfer capacity is the only variation during the second cultivation stage. As soon as the cultures are oxygen-limited, the RQ increases and 2,3-butanediol production is initiated (Fig. 3b, c). Application of the two stage cultivation profile without MES buffer addition results in the same behavior in terms of OTR, RQ, 2,3-butanediol concentration and pH, compared to the respective cultivations with a constant shaking frequency (cf. Fig. 2). Consequently, defined cultivation conditions are obtained without changing the general cultivation characteristics.

As depicted in Fig. 3d, addition of MES buffer prevented acidification of the cultures below pH values of 6 for both filling volumes. Thereby, the early decrease of OTR and RQ for the cultivation with 10 mL filling volume, which was observed without MES buffer addition (Fig. 2), could be prevented. For the cultivations with 20 mL filling volume, similar courses of OTR, RQ and 2,3-butanediol formation are observed irrespective of MES buffer addition.

By the addition of 100 mM MES buffer and application of a two-stage cultivation strategy, defined cultivation conditions were established without changing the general cultivation characteristics. Thereby, the effect of a broad range of different maximum oxygen transfer capacities can be investigated. Consequently, these conditions were applied for all following cultivations.

**Fig. 4:**
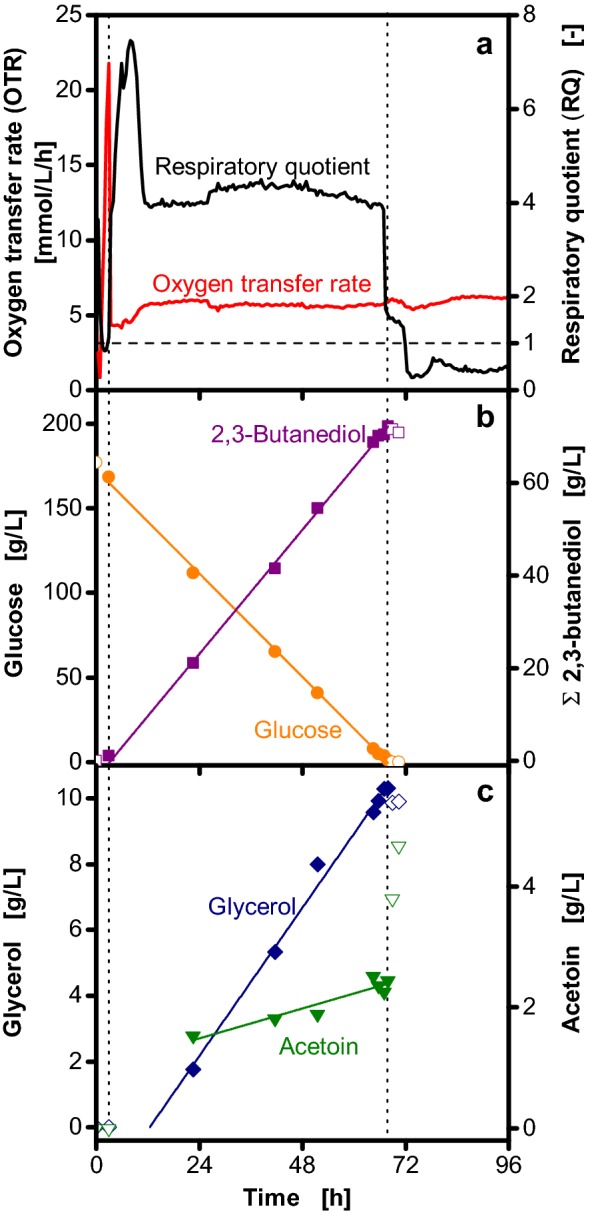
Glucose consumption and product formation during the cultivation of *Bacillus licheniformis* DSM 8785. As indicated by the left vertical dotted line, the shaking frequency was reduced from 350 to 100 rpm after 3 h. Data on oxygen transfer rate (OTR) and respiratory quotient (RQ) (**a**), glucose and total 2,3-butanediol concentration (**b**) and glycerol and acetoin concentration (**c**) are depicted. The solid lines represent linear correlations during the 2,3-butanediol production phase (between the dotted vertical lines) with R^2^ ≥ 0.99 for glucose, 2,3-butanediol and glycerol and R^2^ = 0.89 for acetoin. Filling of symbols represents data points that were (closed symbols) or were not (open symbols) considered for linear correlation. 2,3-Butanediol is shown as sum of the stereoisomers. The RQ is only shown for OTR > 1 mmol/L/h. To account for the increase of metabolite concentrations due to evaporation of water, all concentrations were corrected accordingly and referred to the initial filling volume. Cultivation conditions: 250 mL unbaffled shake flasks, temperature: 37 °C, shaking frequency: 350/100 rpm, shaking diameter: 50 mm, filling volume: 30 mL, Nakashimada medium with 100 mM MES buffer

To determine the maximum 2,3-butanediol concentration, offline sampling was focused on the time of the dropping RQ (Fig. 4). At this point glucose is depleted and the maximum 2,3-butanediol concentration is reached (Fig. 4b). During oxygen limitation, constant glucose consumption and 2,3-butanediol production rates were observed until glucose depletion. For glycerol production a similar trend is visible as for 2,3-butanediol. It is produced at a constant rate, reaches its maximum concentration upon glucose depletion and is consumed afterwards (Fig. 4c). However, in contrast to 2,3-butanediol, glycerol production does not start as soon as oxygen limitation starts, but is delayed by a few hours. The acetoin concentration slowly increases as long as glucose is present, followed by rapid acetoin accumulation upon glucose depletion.

Stoichiometric calculations show that high RQs result from consumption of glucose and formation of reduced products (2,3-butanediol, glycerol and acetoin), whereas low RQs result from the oxidation of 2,3-butanediol to acetoin and the consumption of glycerol. Therefore, online monitoring of the RQ allows to identify this metabolic switching point, which is not visible in OTR or dissolved oxygen measurements. Identification of this switching point is crucial to assess the impact of different cultivation conditions as samples have to be taken exactly at this point to correctly determine the maximum 2,3-butanediol concentration. Therefore, without determination of this time point, very close sampling intervals throughout the cultivation would be required. With the aid of online RQ monitoring fewer samples have to be taken. Still, the requirement to take samples at a distinct time point, which in addition changes with changing cultivation conditions, makes the experiments very complicated. Therefore, a methodology to determine the maximum 2,3-butanediol concentration was developed combining offline sampling and online monitoring of the RQ. The maximum 2,3-butanediol concentration is calculated from the 2,3-butanediol production rate and the time point of glucose depletion, which is determined from the dropping RQ. The 2,3-butanediol production rate is derived from offline samples taken at arbitrary time points during the 2,3-butanediol production phase. With this methodology, the maximum 2,3-butanediol concentration can be calculated for every experiment without the need of sampling at defined time points. Besides 2,3-butanediol, also the concentrations of glucose, glycerol and acetoin can be calculated with this methodology. To verify the basis for this methodology, constant production rates throughout the oxygen-limited phase were confirmed for different maximum oxygen transfer capacities and initial glucose concentrations (Additional file 2). Thereby, the average deviation between the measured and the calculated 2,3-butanediol concentration of the respective experiment was 3.6%.

**Fig. 5:**
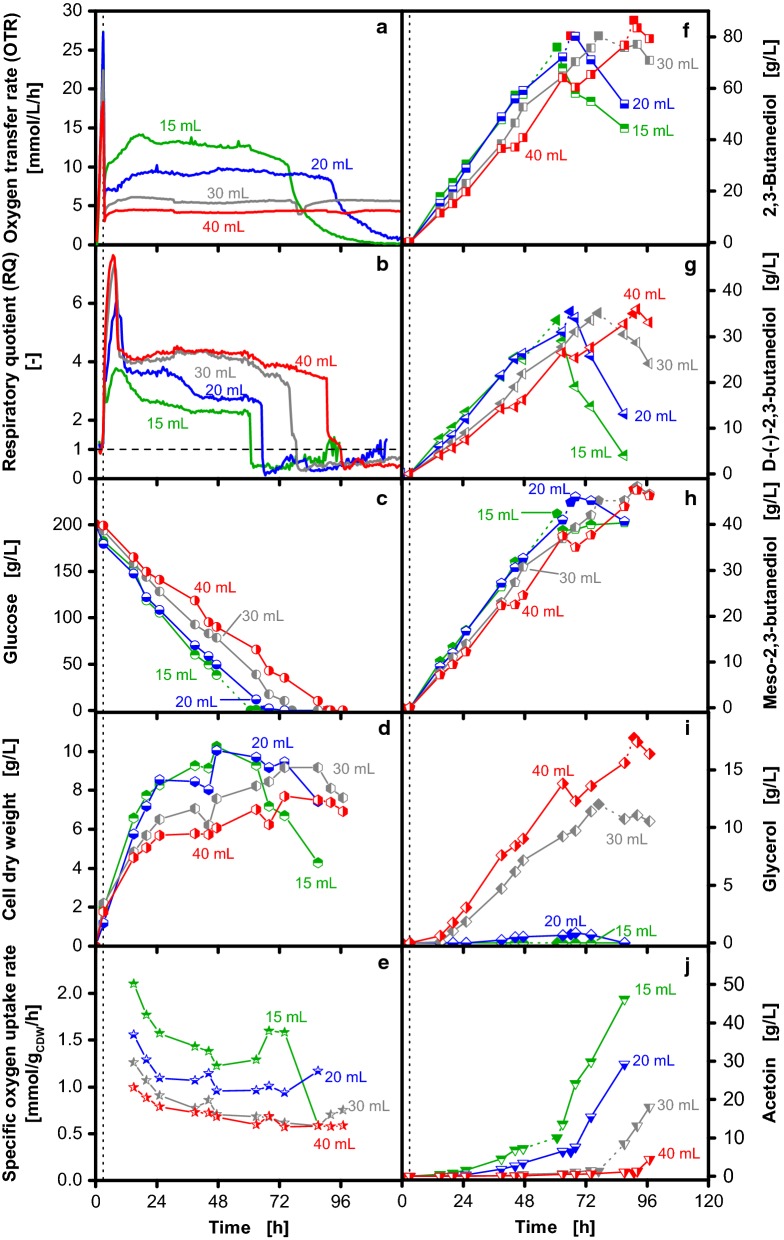
Influence of different maximum oxygen transfer capacities on 2,3-butanediol production with *Bacillus licheniformis* DSM 8785. Variation of the filling volume results in different maximum oxygen transfer capacities in shake flask cultivations. As indicated by the vertical dotted line, the shaking frequency was reduced from 350 to 100 rpm after 3 h. Thereby, oxygen-limited conditions are induced at the same time for all cultivations. Data on oxygen transfer rate (OTR) (**a**), respiratory quotient (RQ) (**b**) glucose (**c**), cell dry weight (**d**), specific oxygen uptake rate (**e**), total 2,3-butanediol (**f**), 2,3-butanediol stereo isomer (**g** and **h**), glycerol (**i**) and acetoin (**j**) concentrations are depicted. In addition to offline samples (half-closed symbols), the concentrations upon glucose depletion were calculated as described in Fig. 4 (closed symbols). For clarity, the connection between the measured and calculated values is shown as dotted line. The RQ is only shown for OTR > 1 mmol/L/h. To account for the increase of metabolite concentrations due to evaporation of water, all concentrations were corrected accordingly and referred to the initial filling volume. Cultivation conditions: 250 mL unbaffled shake flasks, temperature: 37 °C, shaking frequency: 350/100 rpm, shaking diameter: 50 mm, Nakashimada medium with 100 mM MES buffer

Based on the described methodology, the influence of different maximum oxygen transfer capacities on the cultivation of *B. licheniformis* DSM 8785 was investigated (Fig. 5). The courses of OTR and RQ resemble the previous experiments. The application of the two-stage cultivation strategy results in a short standardized oxygen-unlimited growth phase followed by an oxygen-limited phase with decreasing maximum oxygen transfer capacity for increased filling volumes (Fig. 5a). A high maximum oxygen transfer capacity leads to higher glucose consumption rates and consequently earlier glucose depletion. This is also reflected in the course of the RQ (Fig. 5b, c). Measured metabolite concentrations are represented by half-closed symbols and connected with solid lines, whereas the calculated concentrations upon glucose depletion are shown as closed symbols with dotted connection lines (also clearly illustrated in Additional file 3). Increased maximum oxygen transfer capacities result in higher biomass formation with cell dry weights of up to 10 g/L (Fig. 5d). Fast biomass formation during the first 24 h of cultivation is followed by a phase of slowly increasing biomass. Around the time of glucose depletion, the cell dry weight decreases. From the cell dry weight measurements and the online monitored maximum oxygen transfer capacity, specific oxygen uptake rates can be calculated (Fig. 5e). With increased maximum oxygen transfer capacity, also the specific oxygen uptake rate increases. However, in contrast to the maximum oxygen transfer capacity, the specific oxygen uptake rates are not constant, but decrease in the course of the cultivation. As observed before, the 2,3-butanediol production rate correlates with the maximum oxygen transfer capacity. However, lower maximum 2,3-butanediol concentrations occur at high maximum oxygen transfer capacities. This finding could only be revealed by calculating the maximum concentration according to the described methodology (Fig. 5f). As *B. licheniformis* produces two different stereoisomers, d- and meso-2,3-butanediol [37, 47], Fig. 5g and h visualize the impact of the maximum oxygen transfer capacity on the 2,3-butanediol composition. As long as glucose is present, both stereoisomers are produced at constant rates, whereby roughly 1.3 times more meso-2,3-butanediol is formed. Upon glucose depletion, only d-2,3-butanediol is oxidized to acetoin, whereas the concentration of meso-2,3-butanediol remains constant. *B. licheniformis* expresses two different 2,3-butanediol dehydrogenases that convert acetoin to 2,3-butanediol resulting in the formation of the two observed stereoisomers [48]. Consequently, the observed conversion of d-2,3-butanediol indicates that only one of the 2,3-butanediol dehydrogenases catalyzes a reversible reaction.

Both, the glycerol formation rate as well as the maximum concentration increase with decreasing maximum oxygen transfer capacity (Fig. 5i). Up to 18 g/L glycerol are formed at the lowest maximum oxygen transfer capacity (40 mL filling volume), whereas glycerol formation is completely prevented for the cultivation with the highest maximum oxygen transfer capacity (15 mL filling volume). In contrast to that, higher maximum oxygen transfer capacities result in higher acetoin concentrations (Fig. 5j). Whereas acetoin only accumulates after glucose depletion for cultivations with low maximum oxygen transfer capacities (30 and 40 mL filling volume), higher maximum oxygen transfer capacities result in increasing acetoin concentrations throughout the cultivation (15 and 20 mL filling volume). Upon glucose depletion, oxidation of d-2,3-butanediol results in acetoin accumulation. As the course of the acetoin concentration drastically changes upon glucose depletion, this aspect was investigated in detail (Additional file 4). This shows that maximum oxygen transfer capacities above 10 mmol/L/h result in increasing acetoin concentrations before glucose depletion.

**Fig. 6:**
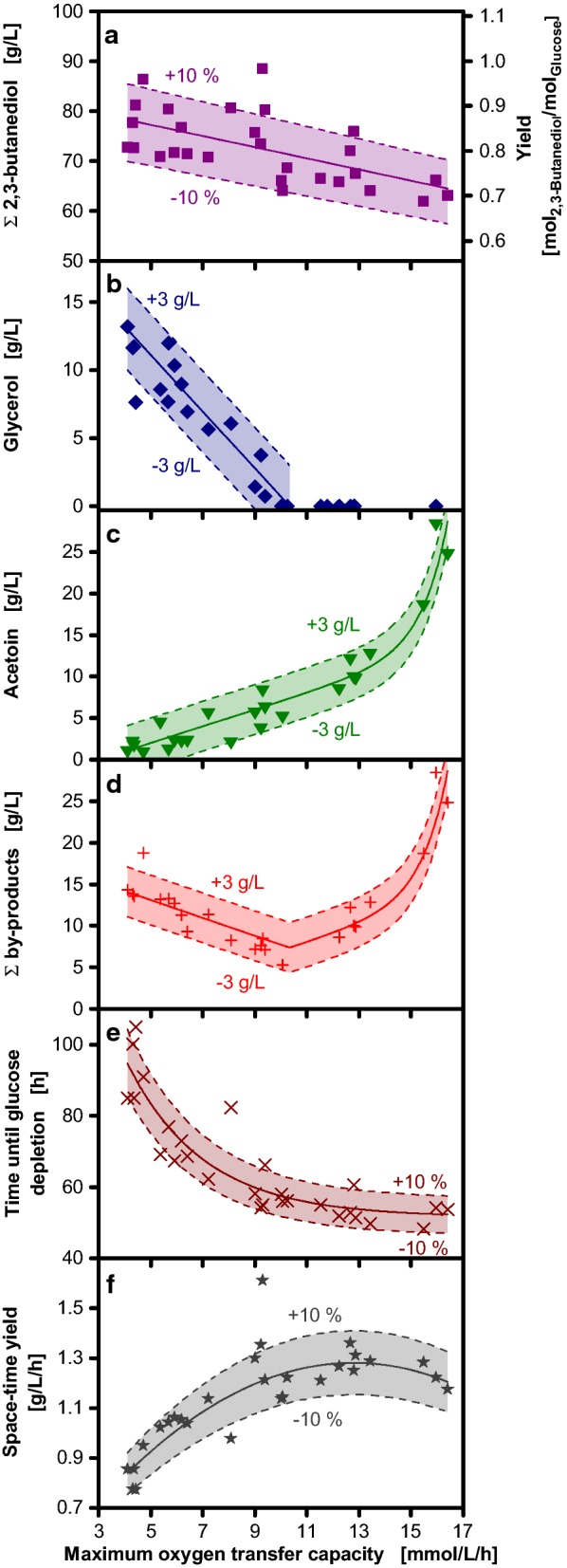
Influence of the maximum oxygen transfer capacity on 2,3-butanediol production with *Bacillus licheniformis* DSM 8785. Calculated maximum total 2,3-butanediol (**a**), glycerol (**b**) and acetoin concentrations (**c**), the sum of accumulated by-products (**d**), the time until glucose was depleted (**e**) and the space–time yield (**f**) are shown in dependency of the maximum oxygen transfer capacity during the 2,3-butanediol production phase. Each data point is derived from one of 28 individual experiments and refers to the time point of glucose depletion. 2,3-Butanediol is shown as sum of the stereoisomers. The time of glucose depletion is determined from the sudden drop of the respiratory quotient, which was measured in biological duplicates. Acetoin, 2,3-butanediol and glycerol concentrations at this time were calculated from offline samples as illustrated in Fig. 4. The space–time yield was calculated from the time of glucose depletion and the maximum 2,3-butanediol concentration. The solid lines represent the fitted linear (**a**, **b**), exponential (**c**, **e**) or cubic (**f**) correlations. The solid line in **e** was calculated as sum of the fitted lines from **b** and **c**. Error margins (10% for **a**, **d** and **e**; 3 g/L for **b**–**d**) are represented by dashed lines. To account for the increase of metabolite concentrations due to evaporation of water, all concentrations were corrected accordingly and referred to the initial filling volume

To analyze the influence of the maximum oxygen transfer capacity in more detail, Fig. 6 shows the calculated metabolite concentrations upon glucose depletion from 28 individual experiments plotted as function of the respective maximum oxygen transfer capacities. Thereby, the observed results can be applied independent of the utilized reactor system and scale. When the maximum oxygen transfer capacity is increased from 4 to 16 mmol/L/h, the maximum 2,3-butanediol concentration linearly decreases from 80 to 65 g/L (Fig. 6a). Consequently, the molar yield also decreases from 0.9 to 0.7, as 180 g/L glucose were used as substrate in all cultivations. This accounts for up to 90% of the stoichiometrically possible yield, neglecting that carbon is also utilized for cell growth or maintenance. The glycerol concentration decreases linearly with increasing maximum oxygen transfer capacity. Glycerol is not formed at all for maximum oxygen transfer capacities higher than 10 mmol/L/h (Fig. 6b). In contrast to that, the acetoin concentration increases with increasing maximum oxygen transfer capacity (Fig. 6c). For maximum oxygen transfer capacities between 4 and 13 mmol/L/h, a moderate increase from 2 to 12 g/L acetoin is observed. However, higher maximum oxygen transfer capacities result in a stronger increase and much higher acetoin concentrations of up to 28 g/L at 16 mmol/L/h. The sum of glycerol and acetoin concentrations is shown in Fig. 6d. A minimum by-product concentration is observed for a maximum oxygen transfer capacity of 10 mmol/L/h resulting from the lowest acetoin concentration without glycerol formation. Consequently, the by-product concentration increases with higher maximum oxygen transfer capacities due to increased acetoin formation. At lower maximum oxygen transfer capacities, higher by-product concentrations result from glycerol formation, which has a stronger impact than the reduced acetoin formation under these conditions.

Apart from product and by-product formation, the overall cultivation time also has a significant impact on the selection of suitable cultivation conditions. For industrial 2,3-butanediol production, cultivations should be terminated as soon as glucose is depleted to achieve the maximum 2,3-butanediol concentration, complete substrate utilization and to avoid acetoin accumulation. Therefore, the influence of the maximum oxygen transfer capacity on the time until glucose is depleted is analyzed in Fig. 6e. Low maximum oxygen transfer capacities of 4 mmol/L/h result in long cultivation times of nearly 100 h. The cultivation time decreases, when the maximum oxygen transfer capacity is increased. Increasing the maximum oxygen transfer capacity above 11 mmol/L/h has only minor effects on the cultivation time. From the cultivation time and the 2,3-butanediol concentration, the space–time yield was calculated (Fig. 6f). The maximum space–time yield of 1.3 g/L/h is reached at a maximum oxygen transfer capacity of 13 mmol/L/h. At higher maximum oxygen transfer capacities, the space–time yield decreases due to lower 2,3-butanediol concentrations. In contrast, lower maximum oxygen transfer capacities lead to decreased space–time yields due to increased cultivation times.

**Fig. 7:**
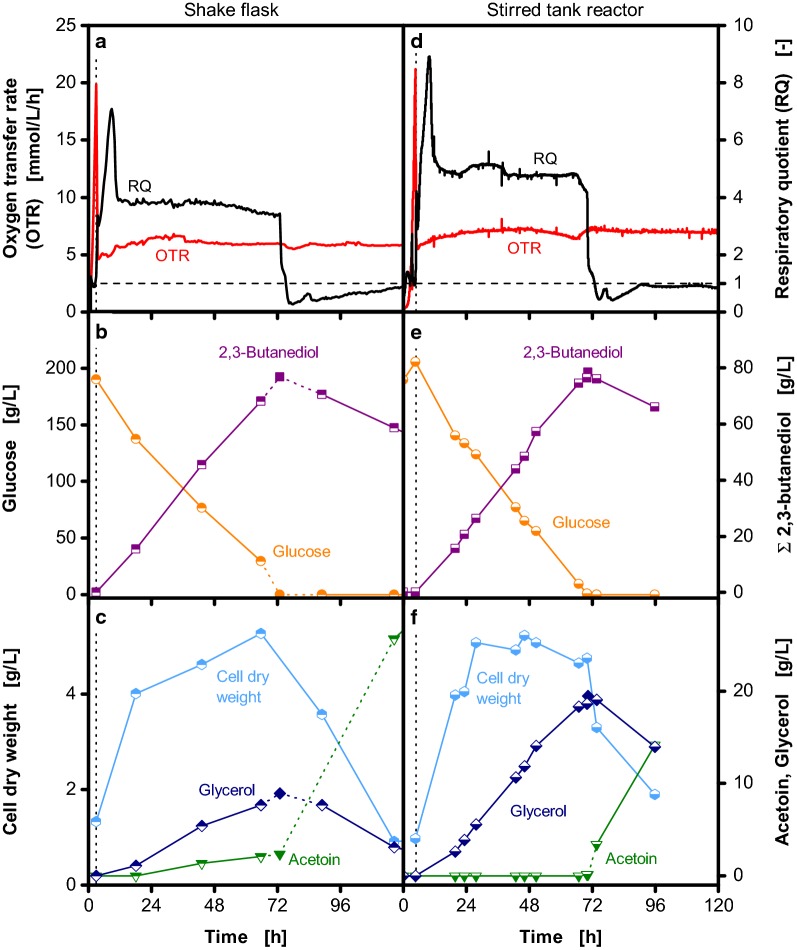
Comparison of shake flask and stirred tank reactor cultivations of *Bacillus licheniformis* DSM 8785. As indicated by the vertical dotted lines, the shaking frequency of the shake flask cultivation (**a**–**c**) and the agitation rate of the stirred tank reactor cultivation (**d**–**f**) were reduced from 350 to 100 and from 1000 to 500 rpm after 4.5 and 3 h, respectively. The courses of oxygen transfer rates (OTR) and respiratory quotients (RQ) (**a**, **d**), glucose and total 2,3-butanediol concentrations (**b**, **e**) as well as cell dry weight, acetoin and glycerol concentrations (**c**, **f**) are shown. In addition to offline samples (half-closed symbols), the concentrations upon glucose depletion were calculated as described in Fig. 4 (closed symbols). For clarity, the connections between the measured and calculated values are shown as dotted lines. 2,3-Butanediol is shown as sum of the stereoisomers. The RQ is only shown for OTR > 1 mmol/L/h. To account for the increase of metabolite concentrations due to evaporation of water, concentrations of the shake flask cultivation were corrected accordingly and referred to the initial filling volume. Cultivation conditions: Temperature: 37 °C; shake flask cultivation: 250 mL unbaffled shake flasks, filling volume: 30 mL, shaking frequency: 350/100 rpm, shaking diameter: 50 mm; stirred tank reactor cultivation: 2 L stirred tank reactor, filling volume: 1.5 L, aeration rate: 0.5 L/min (0.33 vvm), agitation rate: 1000/500 rpm

To verify that the obtained results can be transferred between different bioreactor systems, a shake flask and a stirred tank reactor cultivation are compared in Fig. 7. As shown in Fig. 7a and d, the same maximum oxygen transfer capacity of 7 mmol/L/h was adjusted in both scales after reduction of the shaking frequency and agitation rate, respectively. This results in comparable process performance as demonstrated by the similar courses of glucose and 2,3-butanediol concentrations (Fig. 7b, e). The course of the RQs is comparable (Fig. 7a, d), however, in the stirred tank reactor a higher RQ results from increased glycerol formation compared to the shake flask cultivation (Fig. 7c, f). The cell dry weight increases to 5 g/L during the first 24 h of cultivation in both bioreactor systems, stays constant until glucose is depleted and decreases afterwards (Fig. 7c, f). The previously described rapid accumulation of acetoin upon glucose depletion is also observed in both bioreactors (Fig. 7c, f). This comparison shows that the maximum oxygen transfer capacity can be used to achieve comparable process performance in shake flasks and lab-scale stirred tank reactors.

## Discussion

High 2,3-butanediol concentrations are generally achieved by additional glucose supplementation during the cultivation [10, 11, 15, 26]. Thereby, the 2,3-butanediol production phase is prolonged by maintaining high glucose concentrations. However, this study aimed at the development of a simple and transferable method to investigate the impact of the maximum oxygen transfer capacity on the cultivation and not on optimized 2,3-butanediol production. Thus, a batch operation mode was chosen to minimize the experimental complexity. Nevertheless, reasonable production conditions were applied as a high initial glucose concentration of 180 g/L was chosen resulting in high 2,3-butanediol concentrations of up to 78 g/L (Fig. 6). Selection of suitable maximum oxygen transfer capacities slightly improves 2,3-butanediol production compared to the results Jurchescu et al. [15] achieved in batch experiments with the same organism in a similar cultivation medium (73 g/L 2,3-butanediol at a space–time yield of 0.86 g/L/h). In this work, the same space–time yield was achieved at a maximum oxygen transfer capacity of 4.3 mmol/L/h, resulting in an increased 2,3-butanediol concentration of 77 g/L. A maximum value of 73 g/L 2,3-butanediol was observed at a maximum oxygen transfer capacity of 8.3 mmol/L/h at an increased space–time yield of 1.27 g/L/h.

The influence of the maximum oxygen transfer capacity on the product and by-product spectrum of *B. licheniformis* can be explained based on the NADH/NAD^+^ balance of the organism. During oxygen limitation, only small amounts of NADH can be oxidized in the electron transport chain. Therefore, glycolysis becomes a major source of the organism’s ATP production. There are two options to oxidize the herein produced NADH to NAD^+^, which is crucial to maintain ATP production via glycolysis. One option is the production of reduced overflow metabolites like 2,3-butanediol or glycerol. Additionally, oxidation via the electron transport chain occurs in accordance with the available amount of oxygen. Consequently, increasing amounts of NADH have to be oxidized by overflow metabolite production with decreasing maximum oxygen transfer capacity. For the production of 1 mol 2,3-butanediol from 1 mol of glucose, 2 mol of NADH are obtained during glycolysis, whereas only 1 mol of NADH is oxidized to NAD^+^ during the reduction of acetoin to 2,3-butanediol [2]. For high maximum oxygen transfer capacities, acetoin accumulates, as enough oxygen is available to oxidize more than 1 mol of NADH via the electron transport chain. For low maximum oxygen transfer capacities, the NADH oxidation in the electron transport chain decreases leading to almost complete acetoin reduction to 2,3-butanediol. Moreover, increasing amounts of glycerol are formed. For glycerol formation from glucose, 1 molar equivalent of NADH is oxidized to NAD^+^ without NADH generation. Therefore, glycerol formation can balance out the NAD^+^ deficiency during 2,3-butanediol production at low maximum oxygen transfer capacities.

Based on the results presented in this study, a suitable maximum oxygen transfer capacity for 2,3-butanediol production with *B. licheniformis* DSM 8785 can be selected. However, this study did not aim at presenting an economically feasible production process, but investigated suitable oxygen supply conditions for specific process demands. The results presented in Fig. 6 clearly show that there is not one defined optimum condition for 2,3-butanediol production with *B. licheniformis*. Different parameters become more or less important depending on the overall production chain and economics. The concentrations of 2,3-butanediol, acetoin and glycerol are especially important with regard to the chosen downstream processing. Depending on the demands, either high 2,3-butanediol concentrations, or low by-product concentrations are most beneficial. It is also important to consider, whether efficient separation of acetoin or glycerol from 2,3-butanediol is possible. For example, via distillation acetoin (boiling point at 148 °C) can be separated from 2,3-butanediol (boiling point at 183 °C) together with the process water. However, for glycerol separation (boiling point at 290 °C), a second distillation step would be necessary. Qureshi et al. [49] investigated vacuum membrane distillation for 2,3-butanediol resulting in constantly decreasing 2,3-butanediol to acetoin ratios. Using different solvents for 2,3-butanediol extraction, Anvari et al. [50] did not observe co-extraction of acetoin. Also during anionic extraction of 2,3-butanediol via phenylboronic acid, no acetoin is co-extracted, whereas co-extraction of glycerol was observed [5]. In these cases, avoiding glycerol formation by selection of maximum oxygen transfer capacities above 10 mmol/L/h will most likely be beneficial. Even though the selection of suitable maximum oxygen transfer capacities strongly depends on the specific process demands, some general conclusions can be drawn. Maximum oxygen transfer capacities above 13 mmol/L/h should be avoided. In that case, 2,3-butanediol concentration and space–time yield decrease, while the acetoin concentration severely increases. Also very low maximum oxygen transfer capacities should not be selected, as this only leads to small increases in the 2,3-butanediol concentration at the expense of severely prolonged cultivation times and higher glycerol formation. Selection of a maximum oxygen transfer capacity between 11 and 13 mmol/L/h will be beneficial for most processes. This results in high space–time yields at low by-product formation and completely avoids glycerol formation. In addition to the selection of a suitable oxygen transfer capacity, many other factors are important for industrially relevant production processes. To increase the final 2,3-butanediol concentration, additional glucose supplementation during the cultivation is essential [10, 15]. Additionally, genetic engineering to decrease by-product formation and to enable production of optically pure 2,3-butanediol will be beneficial [12, 51, 52]. Furthermore, the cultivation medium has to be optimized with respect to utilization of cheaper carbon sources and media components [18, 23, 30, 53, 54].

Studies on the effect of the oxygen availability on 2,3-butanediol production revealed different results for different organisms [27, 30, 40, 41]. Therefore, this study focused on the development of a fast and simple, but reliable methodology to investigate this effect. In contrast to the existing studies [27, 30, 35, 40–42], two concepts were applied to increase the precision of the experimental results. First, online monitoring of the respiratory quotient was combined with offline sampling to determine metabolite concentrations exactly at the point of glucose depletion. This prevents misinterpretation of results due to inadequate sampling time points, as 2,3-butanediol and glycerol are consumed and acetoin accumulates after glucose depletion (Fig. 4). To the authors’ knowledge, this effect has not yet been considered in the existing literature. Additionally, a defined two-stage cultivation profile was applied. Two-stage cultivation strategies have already been described in the literature to accelerate 2,3-butanediol formation [11, 14, 33–39]. In contrast, the here applied two-stage cultivation profile aimed at providing defined cultivation conditions instead of accelerating product formation.

Even though the impact of the oxygen availability on 2,3-butanediol production is widely discussed in the literature, only few studies focused on this effect on the basis of scalable parameters [27, 30, 35, 40–42]. However, this is necessary to transfer the obtained results to other bioreactor setups and, thus, make them available to other research groups. Therefore, the maximum oxygen transfer capacity was selected to quantify the effects of oxygen availability in this study. In contrast to the above-mentioned studies, this study presents a shake flask methodology to reduce the technical and experimental effort. The impact of the maximum oxygen transfer capacity on the cultivation (Fig. 6) can be compared to the results Rebecchi et al. [30] generated in a stirred tank reactor with the same organism. The same maximum 2,3-butanediol yields of 0.9 mol/mol (0.45 g/g) were observed in both studies. However, a linear dependency between maximum oxygen transfer capacity and yield was observed in this study, whereas Rebecchi et al. [30] observed reduced yields between 2 and 7 mmol/L/h. This probably results from missing offline samples at the time of maximum 2,3-butanediol concentration. In this study, this problem is avoided by the described method to quantify the maximum 2,3-butanediol concentration. The highest space–time yield was observed at maximum oxygen transfer capacities slightly above 10 mmol/L/h in both studies. However, in this study a higher space–time yield was reached (1.3 instead of 1.0 g/L/h), which probably results from the utilization of different cultivation media. In both studies, exactly the same maximum oxygen transfer capacity of at least 10 mmol/L/h prevented glycerol formation. Generally, the shake flask-based results in this study are in good agreement with the results from stirred tank reactor cultivations. This demonstrates that this is a suitable approach to investigate the impact of the maximum oxygen transfer capacity on 2,3-butanediol production.

## Conclusion

A fast and precise shake flask methodology to determine the impact of the oxygen availability on 2,3-butanediol production was developed. Thereby, optimum cultivation conditions for *B. licheniformis* DSM 8785 were identified at maximum oxygen transfer capacities between 11 and 13 mmol/L/h. Under these conditions, high product and space–time yields are achieved, while glycerol formation is completely prevented and only small amounts of acetoin accumulate.

The application of a defined two-stage cultivation strategy synchronizes the initiation of oxygen limitation between different cultivations and, thus, allows for the isolated determination of the effects of the maximum oxygen transfer capacity. Additionally, a combination of online monitoring of the respiratory quotient and offline sampling provides an experimentally easy way to determine the maximum 2,3-butanediol concentration, as 2,3-butanediol is consumed upon glucose depletion. Thereby, wrong conclusions resulting from an offset between the sampling times and the time of maximum 2,3-butanediol concentration are prevented.

As the oxygen availability is investigated based on the maximum oxygen transfer capacity, the experimental results can easily be transferred to other bioreactor types and setups. Compared to the already existing stirred tank reactor-based approaches, the here presented shake flask methodology leads to similar results at reduced experimental effort and costs.

## Methods

### Microorganism and medium composition

All experiments were performed with *Bacillus licheniformis* DSM 8785. The cells were stored at − 80 °C in 150 g/L glycerol. Pre- and main-cultures were performed in a complex medium based on the medium described by Nakashimada et al. [55]. The medium contained 180 g/L glucose, 5 g/L yeast extract (Karl Roth GMBH, Karlsruhe, Germany), 5 g/L tryptone (Karl Roth GMBH, Karlsruhe, Germany), 7 g/L K_2_HPO_4_, 5.5 g/L KH_2_PO_4_, 1 g/L (NH_4_)_2_SO_4_, 0.25 g/L MgSO_4_·7H_2_O, 0.12 g/L Na_2_MoO_4_·2H_2_O, 0.021 g/L CaCl_2_·2H_2_O, 0.029 g/L Co(NO_3_)_2_·6H_2_O, 0.039 g/L (NH_4_)_2_Fe(SO_4_)_2_·6H_2_O, 0.002 g/L nicotinic acid, 0.0002 g/L Na_2_SeO_3_, 0.00005 g/L NiCl_2_·6H_2_O, 0.005 g/L MnCl_2_·4H_2_O, 0.001 g/L H_3_BO_3_, 0.0002 g/L AlK(SO_4_)_2_·12H_2_O, 0.00001 g/L CuCl_2_·2H_2_O, and 0.0055 g/L Na_2_EDTA·2H_2_O. To the described medium, 100 mM MES (2-(*N*-morpholino)ethanesulfonic acid) buffer was added to prevent acidification during the cultivation.

### Shake flask cultivations

Cultivations were performed in unbaffled 250 mL shake flasks on an orbital shaker (Climo shaker ISF1-X, Adolf Kühner AG, Birsfelden, Switzerland), with a shaking diameter of 50 mm at 37 °C. For the pre-culture, 20 mL medium per flask were inoculated with 20 µL from the glycerol stock, cultivated at 350 rpm and harvested in the exponential growth phase that was determined via online monitoring of the OTR. For the main culture, a master mix was inoculated from the pre culture to an initial optical density (OD_600_) of 0.1. The master mix was then distributed to the individual shake flasks. At the beginning of the cultivation, a shaking frequency of 350 rpm was adjusted, which was reduced to 100 rpm when an OTR of 20 mmol/L/h was reached. As visualized in Fig. 1, online monitoring of the respiratory activity was combined with offline sampling. All shake flasks were cultivated in parallel under identical conditions. For offline sampling, conventional 250 mL shake flasks were used. At each sampling point, one flask for each cultivation condition was removed from the shaker, subjected to offline analysis and not returned to the shaker again. Online monitoring was performed with an in-house fabricated Respiration Activity Monitoring System (RAMOS) [45]. Commercial versions of the RAMOS device are available from HiTec Zang GMBH (Herzogenrath, Germany) or Adolf Kühner AG (Birsfelden, Switzerland).

### Stirred tank reactor cultivations

A 2 L Autoclavable Bioreactor (Applikon Biotechnology, Foster City, USA) was equipped with three baffles and two Rushton Turbines (6 blades, 45 mm stirrer diameter) and operated with a filling volume of 1.5 L. The reactor was aerated with 0.5 L/min (0.33 vvm) pressurized air via an L-type gas sparger. The temperature was adjusted to 37 °C using a Pt-100 sensor (Applikon Biotechnology, Foster City, USA) and the integrated cooling jacket. An X-STREAM exhaust gas analyzer (Emerson Process Management GmbH, Wessling, Germany) was used to obtain OTR, CTR and RQ. Before inoculation, Plurafac LF 1300 anti-foaming agent (BASF, Ludwigshafen, Germany) was added (2 mL). 20 mL of an overnight pre-culture (cultivation conditions as described for the shake flask experiments) were used for inoculation. For the first 4.5 h, an agitation rate of 1000 rpm was used to maintain oxygen unlimited conditions, which was then reduced to 500 rpm to initiate oxygen limitation. In contrast to shake flask cultivations, no MES buffer was added. Detrimental acidification was prevented by automated addition of 2 M NaOH when the pH dropped below 5.5.

### Offline analytics

Upon sampling, the remaining liquid volume in the individual shake flasks was determined, resulting in an evaporation rate of 0.04 mL/h. At the point of maximum 2,3-butanediol concentration, between 7 and 20% of the initial filling volume have evaporated (for cultivations with 60 and 10 mL filling volume, respectively). To account for the increase of metabolite concentrations due to this evaporation of water, all concentrations were corrected accordingly and referred to the initial filling volume. In stirred tank reactor cultivations, evaporation was minimized using an exhaust gas cooler and not considered for offline analysis. Cells were separated via centrifugation (5 min at 17,968*g*) and the cell dry weight (dried for at least 2 days at 60 °C) was determined as duplicates after two washing steps with deionized water in pre-dried 2 mL reaction tubes. The supernatant of the first centrifugation step was used for further analysis. Metabolite concentrations of the filtered supernatant were determined via high-performance liquid chromatography (HPLC) after 20-fold dilution with deionized water. Glucose, 2,3-butanediol, acetoin and glycerol were quantified with a Dionex UltiMate 3000 HPLC system (Dionex, Sunnyvale, USA) at 70 °C using an organic acid-resin (250·8 mm, CS-Chromatographie, Langerwehe, Germany) and a Shodex RI-101 detector (Showa Denko, Munich, Germany). As mobile phase 2.5 mM sulfuric acid was chosen with a flow rate of 0.5 mL/min. Unless specified otherwise, 2,3-butanediol concentrations in this work always refer to the sum of all stereoisomers. With the applied method, meso-2,3-butanediol was separated from the d- and l-isomer, whereas distinction between the latter was not possible. As only production of meso- and d-2,3-butanediol is described in the literature for *B. licheniformis* [37, 47], the second peak was considered to be d-2,3-butanediol. The pH was measured with a CyberScan pH 510 (Eutech Instruments, Landsmeer, The Netherlands) pH meter.

### Calculation of metabolite concentrations upon glucose depletion

A combination of offline sampling and online monitoring of the respiratory activity was used to calculate the concentrations of 2,3-butanediol, acetoin and glycerol upon glucose depletion. The time point of glucose depletion was determined based on the sudden drop of the RQ as mean of duplicate cultivations (cf. Fig. 4). A decrease of more than 20% between two consecutive measurement points was chosen as cut-off value. The corresponding concentration was calculated from a linear correlation derived from offline samples that were taken during the oxygen-limited phase and before the point of glucose depletion. Only samples with concentrations > 0 g/L of the respective metabolite were considered.

## Additional files

**Additional file 1.** Reduced acidification during the cultivation of *Bacillus licheniformis* DSM 8785. Cultivations in 10 mL Nakashimada medium with addition of different MES buffer concentrations (0, 100 and 200 mM as indicated in the figure) and different initial pH values (6.5 and 7.0 as indicated in the figure) are compared. Without addition of MES buffer, acetate formation was observed (up to 4.0 and 5.8 g/L for the cultivations with initial pH of 6.5 and 7, respectively). No acetate was measured in the cultures with MES addition. As indicated by the vertical dotted line, the shaking frequency was reduced from 350 to 100 rpm after 3 h. Thereby, oxygen-limited conditions are induced at the same time for all cultivations. Data on oxygen transfer rate (OTR) (**a**), glucose and (**b**) total 2,3-butanediol concentration (**c**) and pH (**d**) are depicted. 2,3-Butanediol is shown as sum of the stereoisomers. To account for the increase of metabolite concentrations due to evaporation of water, all concentrations were corrected accordingly and referred to the initial filling volume. Cultivation conditions: 250 mL unbaffled shake flasks, temperature: 37 °C, shaking frequency: 350/100 rpm, shaking diameter: 50 mm, filling volume: 10 mL.

**Additional file 2.** Glucose consumption and product formation during different cultivations of *Bacillus licheniformis* DSM 8785. Cultivations with varied filling volumes and glucose concentrations are compared as described in the figure legend. Variation of the filling volume results in different maximum oxygen transfer capacities in shake flask cultivations. The shaking frequency was reduced from 350 to 100 rpm after 3 h. Thereby, oxygen-limited conditions are induced at the same time for all cultivations. Data on glucose (**a**) total 2,3-butanediol (**b**) glycerol (**c**) and acetoin concentration (**d**) are depicted. 2,3-Butanediol is shown as sum of the stereoisomers. The vertical dotted lines represent the time of glucose depletion derived from the sharply decreasing respiratory quotient (data not shown) as described in Fig. 4. At this point the average deviation between the calculated and measured 2,3-butanediol concentration was 3.6% (2.4, 8.8, 0.7, 3.4 and 2.7% for the individual cultivations from top to bottom of the legend). To account for the increase of metabolite concentrations due to evaporation of water, all concentrations were corrected accordingly and referred to the initial filling volume. Cultivation conditions: 250 mL unbaffled shake flasks, temperature: 37 °C, shaking frequency: 350/100 rpm, shaking diameter: 50 mm, Nakashimada medium with 100 mM MES buffer (90 or 180 g/L glucose).

**Additional file 3.** 2,3-Butanediol formation and subsequent consumption during the cultivation of *Bacillus licheniformis* DSM 8785. As indicated by the vertical dotted line, the shaking frequency was reduced from 350 to 100 rpm after 3 h. Data on oxygen transfer rate (OTR) and respiratory quotient (RQ) (**a**), and total 2,3-butanediol and glucose concentration (**b**) are depicted. In addition to offline samples (open symbols), the concentrations upon glucose depletion were calculated as illustrated in Fig. 4 (closed symbols). For clarity, the connection between the measured and calculated values is shown as dotted line. 2,3-Butanediol is shown as sum of the stereoisomers. The RQ is only shown for OTR > 1 mmol/L/h. To account for the increase of metabolite concentrations due to evaporation of water, all concentrations were corrected accordingly and referred to the initial filling volume. Cultivation conditions: 250 mL unbaffled shake flasks, temperature: 37 °C, shaking frequency: 350/100 rpm, shaking diameter: 50 mm, filling volume: 30 mL, Nakashimada medium with 100 mM MES buffer.

**Additional file 4.** Influence of maximum oxygen transfer capacities on acetoin formation with *Bacillus licheniformis* DSM 8785. Variation of the filling volume results in different maximum oxygen transfer capacities in shake flask cultivations. The shaking frequency was reduced from 350 to 100 rpm after 3 h. Thereby, oxygen-limited conditions are induced at the same time for all cultivations. Oxygen transfer rates (OTR) (**a**) and acetoin concentrations (**b**) are depicted. The time on the x-axis is presented relative to the time of glucose depletion (0 h). In addition to offline samples (open symbols), the concentrations upon glucose depletion were calculated as described in Fig. 4 (closed symbols). For clarity, the connection between the measured and calculated values is shown as dotted line. The time of glucose depletion is derived from the sudden drop of the respiratory quotient (RQ), as illustrated in Fig. 4. To account for the increase of metabolite concentrations due to evaporation of water, all concentrations were corrected accordingly and referred to the initial filling volume. Cultivation conditions: 250 mL unbaffled shake flasks, temperature: 37 °C, shaking frequency: 350/100 rpm, shaking diameter: 50 mm, Nakashimada medium with 100 mM MES buffer.

## Abbreviations

MES: (2-(*N*-morpholino)ethanesulfonic acid)
OTR: oxygen transfer rate
CTR: carbon dioxide transfer rate
RQ: respiratory quotient
HPLC: high-performance liquid chromatography
