# Supplementary figures and images for: Shake flask methodology for assessing the influence of the maximum oxygen transfer capacity on 2,3-butanediol production
Source: Microb Cell Fact. 2019 May 3;18:78. doi: 10.1186/s12934-019-1126-9 (PMC6498610; doi:10.1186/s12934-019-1126-9)

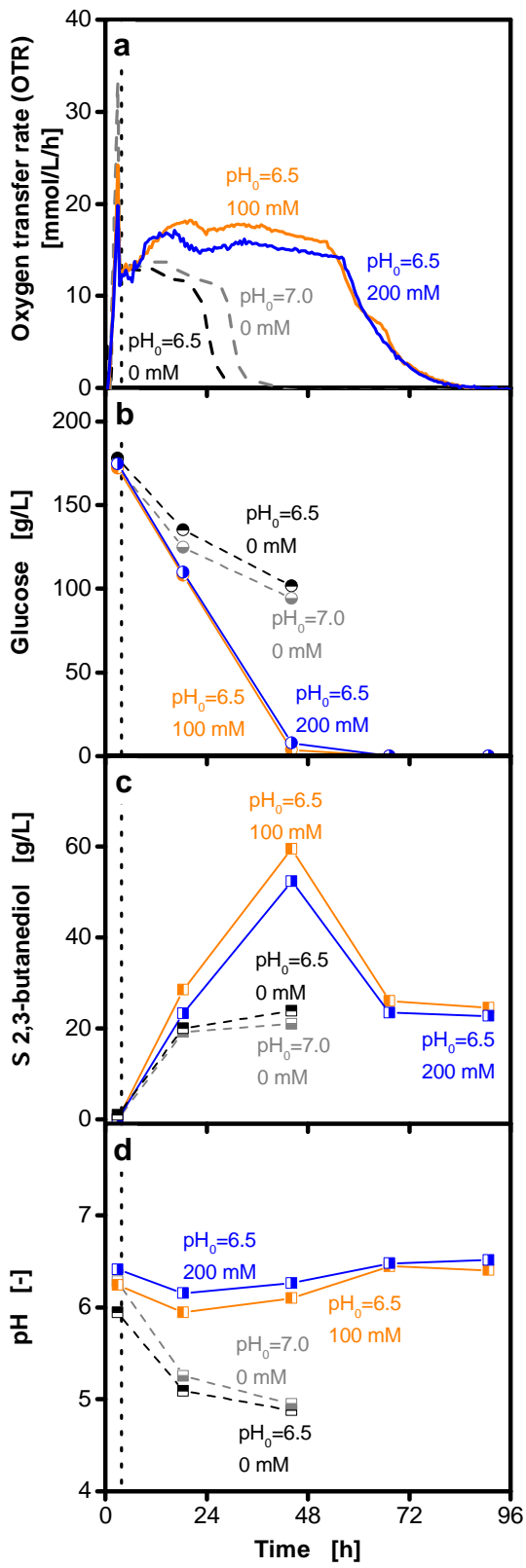

Supplement: Supplementary file 1 — Additional file 1. Reduced acidification during the cultivation of Bacillus licheniformis DSM 8785. Cultivations in 10 mL Nakashimada medium with addition of different MES buffer concentrations (0, 100 and 200 mM as indicated in the figure) and different initial pH values (6.5 and 7.0 as indicated in the figure) are compared. Without addition of MES buffer, acetate formation was observed (up to 4.0 and 5.8 g/L for the cultivations with initial pH of 6.5 and 7, respectively). No acetate was measured in the cultures with MES addition. As indicated by the vertical dotted line, the shaking frequency was reduced from 350 to 100 rpm after 3 h. Thereby, oxygen-limited conditions are induced at the same time for all cultivations. Data on oxygen transfer rate (OTR) (a), glucose and (b) total 2,3-butanediol concentration (c) and pH (d) are depicted. 2,3-Butanediol is shown as sum of the stereoisomers. To account for the increase of metabolite concentrations due to evaporation of water, all concentrations were corrected accordingly and referred to the initial filling volume. Cultivation conditions: 250 mL unbaffled shake flasks, temperature: 37 °C, shaking frequency: 350/100 rpm, shaking diameter: 50 mm, filling volume: 10 mL. [file 12934_2019_1126_MOESM1_ESM.pdf]

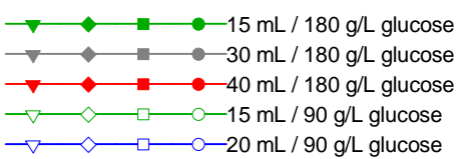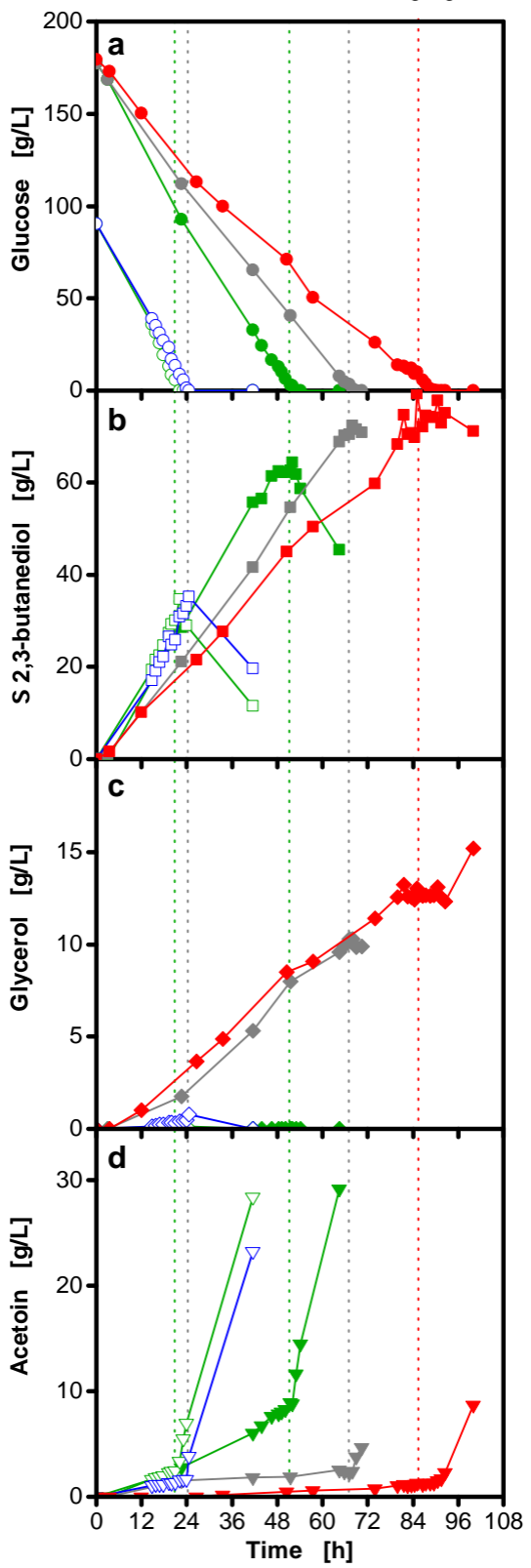

Supplement: Supplementary file 2 — Additional file 2. Glucose consumption and product formation during different cultivations of Bacillus licheniformis DSM 8785. Cultivations with varied filling volumes and glucose concentrations are compared as described in the figure legend. Variation of the filling volume results in different maximum oxygen transfer capacities in shake flask cultivations. The shaking frequency was reduced from 350 to 100 rpm after 3 h. Thereby, oxygen-limited conditions are induced at the same time for all cultivations. Data on glucose (a) total 2,3-butanediol (b) glycerol (c) and acetoin concentration (d) are depicted. 2,3-Butanediol is shown as sum of the stereoisomers. The vertical dotted lines represent the time of glucose depletion derived from the sharply decreasing respiratory quotient (data not shown) as described in Fig. 4. At this point the average deviation between the calculated and measured 2,3-butanediol concentration was 3.6% (2.4, 8.8, 0.7, 3.4 and 2.7% for the individual cultivations from top to bottom of the legend). To account for the increase of metabolite concentrations due to evaporation of water, all concentrations were corrected accordingly and referred to the initial filling volume. Cultivation conditions: 250 mL unbaffled shake flasks, temperature: 37 °C, shaking frequency: 350/100 rpm, shaking diameter: 50 mm, Nakashimada medium with 100 mM MES buffer (90 or 180 g/L glucose). [file 12934_2019_1126_MOESM2_ESM.pdf]

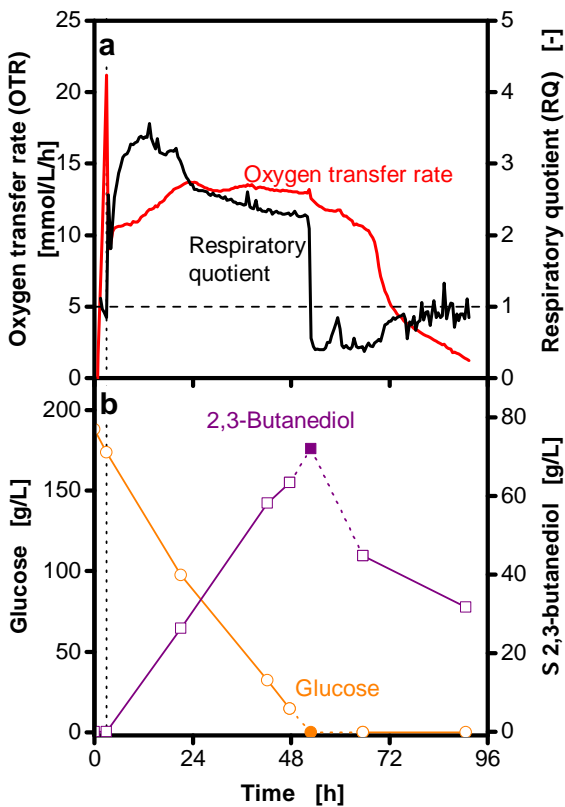

Supplement: Supplementary file 3 — Additional file 3. 2,3-Butanediol formation and subsequent consumption during the cultivation of Bacillus licheniformis DSM 8785. As indicated by the vertical dotted line, the shaking frequency was reduced from 350 to 100 rpm after 3 h. Data on oxygen transfer rate (OTR) and respiratory quotient (RQ) (a), and total 2,3-butanediol and glucose concentration (b) are depicted. In addition to offline samples (open symbols), the concentrations upon glucose depletion were calculated as illustrated in Fig. 4 (closed symbols). For clarity, the connection between the measured and calculated values is shown as dotted line. 2,3-Butanediol is shown as sum of the stereoisomers. The RQ is only shown for OTR > 1 mmol/L/h. To account for the increase of metabolite concentrations due to evaporation of water, all concentrations were corrected accordingly and referred to the initial filling volume. Cultivation conditions: 250 mL unbaffled shake flasks, temperature: 37 °C, shaking frequency: 350/100 rpm, shaking diameter: 50 mm, filling volume: 30 mL, Nakashimada medium with 100 mM MES buffer. [file 12934_2019_1126_MOESM3_ESM.pdf]

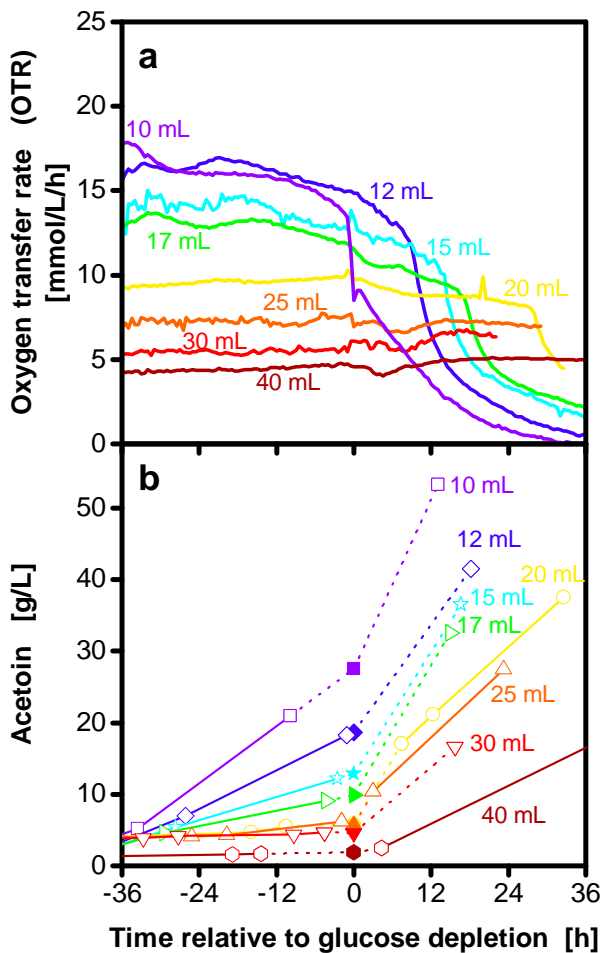

Supplement: Supplementary file 4 — Additional file 4. Influence of maximum oxygen transfer capacities on acetoin formation with Bacillus licheniformis DSM 8785. Variation of the filling volume results in different maximum oxygen transfer capacities in shake flask cultivations. The shaking frequency was reduced from 350 to 100 rpm after 3 h. Thereby, oxygen-limited conditions are induced at the same time for all cultivations. Oxygen transfer rates (OTR) (a) and acetoin concentrations (b) are depicted. The time on the x-axis is presented relative to the time of glucose depletion (0 h). In addition to offline samples (open symbols), the concentrations upon glucose depletion were calculated as described in Fig. 4 (closed symbols). For clarity, the connection between the measured and calculated values is shown as dotted line. The time of glucose depletion is derived from the sudden drop of the respiratory quotient (RQ), as illustrated in Fig. 4. To account for the increase of metabolite concentrations due to evaporation of water, all concentrations were corrected accordingly and referred to the initial filling volume. Cultivation conditions: 250 mL unbaffled shake flasks, temperature: 37 °C, shaking frequency: 350/100 rpm, shaking diameter: 50 mm, Nakashimada medium with 100 mM MES buffer. [file 12934_2019_1126_MOESM4_ESM.pdf]
